# Neural substrates of sexual arousal are not sex dependent

**DOI:** 10.1073/pnas.1904975116

**Published:** 2019-07-15

**Authors:** Ekaterina Mitricheva, Rui Kimura, Nikos K. Logothetis, Hamid R. Noori

**Affiliations:** ^a^Department of Physiology of Cognitive Processes, Max Planck Institute for Biological Cybernetics, 72076 Tübingen, Germany;; ^b^Imaging Science and Biomedical Engineering, University of Manchester, Manchester M13 9PL, United Kingdom

**Keywords:** sexual arousal, neuroimaging, sex differences, metaanalysis

## Abstract

Sexual arousal is a dynamical, highly coordinated neurophysiological process that is often induced by visual stimuli. Numerous studies have proposed that the cognitive processing stage of responding to sexual stimuli is the first stage, in which sex differences occur, and the divergence between men and women has been attributed to differences in the concerted activity of neural networks. The present comprehensive metaanalysis challenges this hypothesis and provides robust quantitative evidence that the neuronal circuitries activated by visual sexual stimuli are independent of biological sex. Sixty-one functional magnetic resonance imaging studies (1,850 individuals) that presented erotic visual stimuli to men and women of different sexual orientation were identified. Coordinate-based activation likelihood estimation was used to conduct metaanalyses. Sensitivity and clustering analyses of averaged neuronal response patterns were performed to investigate robustness of the findings. In contrast to neutral stimuli, sexual pictures and videos induce significant activations in brain regions, including insula, middle occipital, anterior cingulate and fusiform gyrus, amygdala, striatum, pulvinar, and substantia nigra. Cluster analysis suggests stimulus type as the most, and biological sex as the least, predictor for classification. Contrast analysis further shows no significant sex-specific differences within groups. Systematic review of sex differences in gray matter volume of brain regions associated with sexual arousal (3,723 adults) did not show any causal relationship between structural features and functional response to visual sexual stimuli. The neural basis of sexual arousal in humans is associated with sexual orientation yet, contrary to the widely accepted view, is not different between women and men.

## Significance

Neuroimaging studies suggest differences in the underlying biology of sexual arousal associated with sex and sexual orientation, yet their findings are conflicting. Following a thorough statistical review of all significant neuroimaging studies, we offer strong quantitative evidence that the neuronal response to visual sexual stimuli, contrary to the widely accepted view, is independent of biological sex. Both men and women show increased activation in many cortical and subcortical brain regions thought to be involved in the response to visual sexual stimuli, while the limited sex differences that have been found and reported previously refer to subjective rating of the content.

Sexual activity is a natural, goal-directed behavior for all species and genders (1), while arousal and desire are often expressed in different ways in men and women. In particular, it is widely assumed that there are sex differences in response to visual sexual stimuli (2) that lead to a larger sexual arousal in men than in women (3–6). A review of these studies (7) proposes that the sexual duality is due to differences in neural information processing, that in turn lead to dimorphisms in physiological response and subjective ratings of arousal. In general, sexual arousal comprises 2 components: genital arousal and subjective (neuronal) arousal (8). The former is characterized by genital vasodilatation and various physiological changes that occur in response to sexual stimuli, whereas the latter refers to the mental engagement during sexual activity. However, the underlying neurobiological mechanisms remain elusive. Sexual dimorphisms of adult brain volumes have been reported in various brain regions, including frontomedial cortex, amygdala, and hypothalamus (9, 10); however, the findings are to some extent inconsistent and even contradictory (11). Despite potential anatomical sex differences, it remains critical to investigate whether brain processes differ at a functional level. With the aim of exploring the neural correlates of sexual arousal as elicited by external sexual stimuli consisting of still images or videos, several neuroimaging studies have investigated the cerebral activation during erotic visual stimulation. In line with the well-documented assumption that sexual stimuli elicit sexual arousal, in the present study we aimed at testing the common supposition of sex differences at neuronal level in an empirical, evidence-based manner and determining whether there are brain networks that show sex-specific response patterns to sexual cues. To this end, we performed a quantitative metaanalysis of all published functional magnetic resonance imaging (fMRI) studies that presented erotic visual stimuli to male and female subjects of different sexual orientation (i.e., bisexual, homosexual, heterosexual, and transsexuals), and compared the metabolically up-regulated neural networks with respect to sex to assess the correspondence of neural activation across multiple neuroimaging studies. Specifically, by means of the so-called activation likelihood estimation (ALE) approach (12, 13), we explored statistically significant concordance of activated voxels across numerous studies while controlling for accidental chance clustering. ALE tests for statistically reliable clustering of activations in standardized locations, avoiding spatial distinction errors and problematic incongruence of labeling across studies that could have befallen narrative-based reviews and tabular metaanalytic approaches on cue-induced sexual arousal. In addition, we conducted a systematic review of sex differences in gray matter volume of brain regions associated with sexual arousal, to elucidate to what extent structural features relate to functional response profiles in men and women.

## Results

### Individual Study and Overall Estimates.

**Fig. 1.**
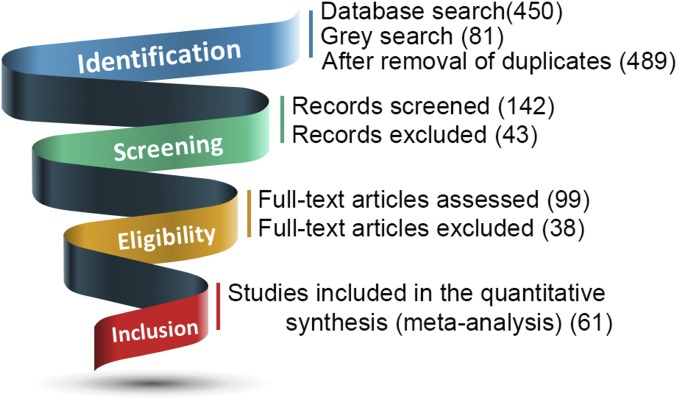
Flow diagram of literature search and study selection.

For the metaanalysis of functional response to visual sexual stimuli, searches of the electronic databases identified 450 unique abstracts, titles, or both as original publications. The gray literature search (i.e., searching the reference sections of identified papers, reviews, and metaanalyses) provided 81 additional articles. A total of 142 research articles were screened and 99 proved potentially relevant for full-text review. Sixty-one articles fulfilled inclusion criteria and were selected for the metaanalysis. A flow diagram of the study selection process is represented in Fig. 1 (detailed chart in *SI Appendix*, Fig. S1). In total, these studies comprised data from 1,850 participants (*SI Appendix*, Table S1). On average, 71.7 ± 45.6% of the cases were male individuals (mean age: 30.0 ± 5.2 y) without history of psychiatric disorders. Sexual orientation was assessed by average Kinsey scale (2.8 ± 0.3 for bisexuals; 5.7 ± 0.5 for homosexuals, and 0.4 ± 0.3 for heterosexuals). Handedness was reported in 63% of studies, among which all participants were right handed. No studies reported 1) the inclusion of subjects with a psychiatric or neurological comorbidity or 2) the inclusion of subjects with current psychotropic medication use, though some studies did not report this information. All of the studies used visual cues, primarily pictures or videos, presented in block or event-related design. Studies with other types of cues (audio, olfactory, etc.) or studies with resting state were excluded.

### Representativeness of the Populations.

All participants were adult (>18 y) healthy volunteers with no history of psychiatric disorders from European, North American, and Asian countries (Germany, Spain, Sweden, United States, Canada, South Korea, and China), while no conclusive deductions could be made with respect to ethnicity.

### Sensitivity Analysis.

In general, no individual study affected the overall statistics. As suggested by the linear regression analyses, the number of significant clusters comprising the neural response patterns correlates positively (Pearson correlation coefficient = 0.54) with the number of reported foci in each study. In contrast, the topology of the neuronal response patterns indicated by the number of significant clusters shows almost no correlation (Pearson correlation coefficient = 0.13) with the sample size of individual studies. This fact indicates that the underlying data for the metaanalysis are representative.

**Fig. 2.**
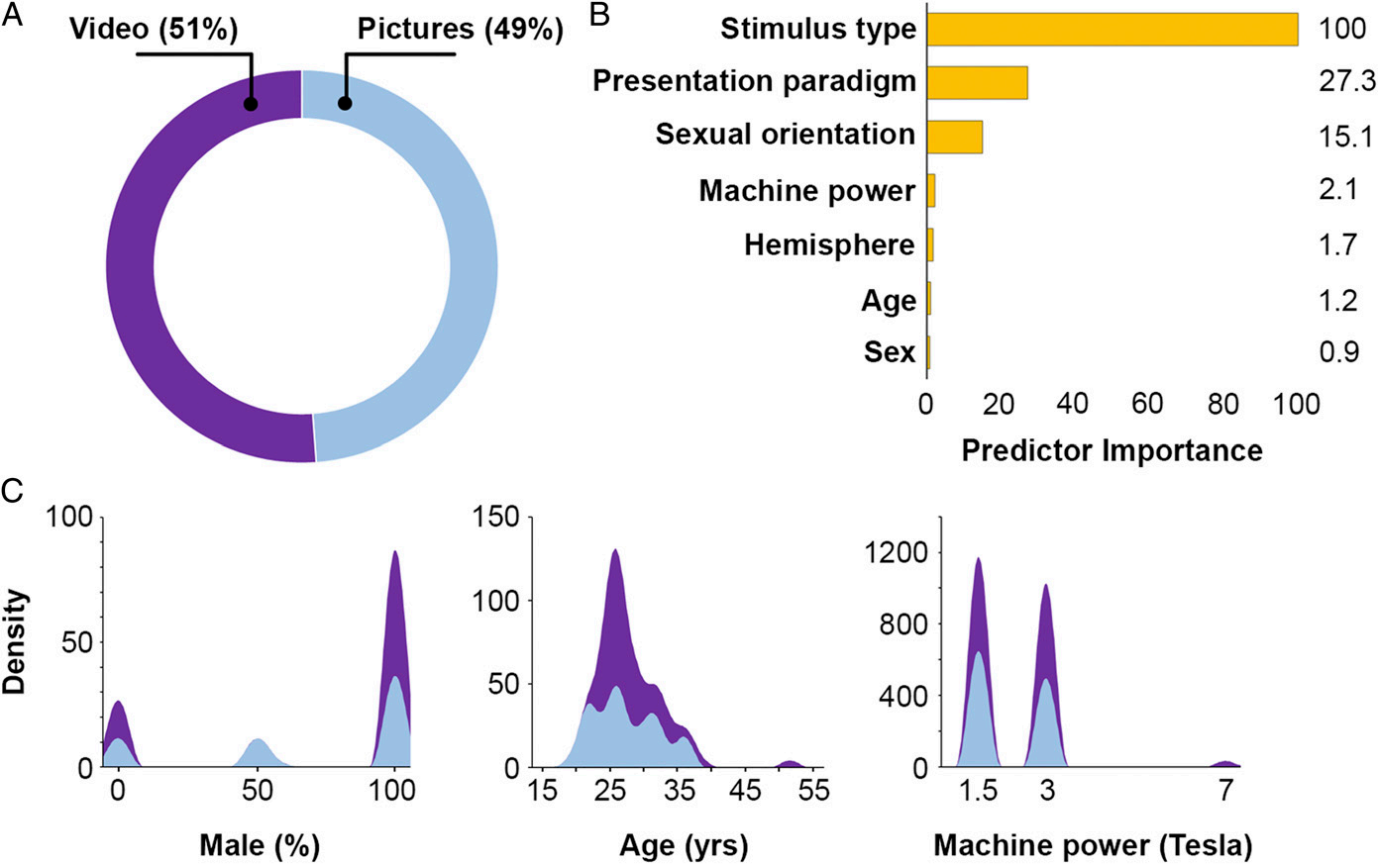
Two-step clustering identifies groupings of categorical and continuous variables by running preclustering first and then by running hierarchical methods. Two clusters of approximately equal size could be obtained (*A*) which related to stimulus type (i.e., picture or video). Presentation paradigm (i.e., block design or event related) and sexual orientation were found to be weak predictors for classifications (*B*), while continuous variables such as machine power, age, or biological sex of study participants showed similar density distributions in both clusters (*C*).

Numerous comparative studies have analyzed the impact of effect modifiers such as handedness (14), presentation design (15), and strength of magnetic field (16) in the context of sexual arousal and orientation. Sexual orientation has been hypothesized to have an early genesis involving neurodevelopment. Because of its early development, neurodevelopmental correlates, and sex dimorphism, handedness can potentially provide more direct and more compelling information on the early neurodevelopmental basis of sexual orientation. Out of 61 included studies, in 39 the participants were right handed, while the remaining publications did not report on handedness. Thus, a reliable sensitivity analysis with respect to handedness was not possible. However, it is noteworthy that a metaanalysis by Lalumière et al. (14) found a significant relationship between handedness and sexual orientation in both sexes, albeit stronger in women. For other effect modifiers, the 2-step hierarchical clustering suggests that stimulus type (i.e., pictures or videos) was the predominant classifier (Fig. 2 *A* and *B*). While the clusters showed sensitivity to presentation paradigm (i.e., block design vs. event related) and sexual orientation, factors such as strength of magnetic field (i.e., machine power in Tesla), lateralization, age and biological sex of participants did not have any significant impact on the clusters (Fig. 2*C*).

### Neural Correlates of Sexual Arousal.

**Fig. 3.**
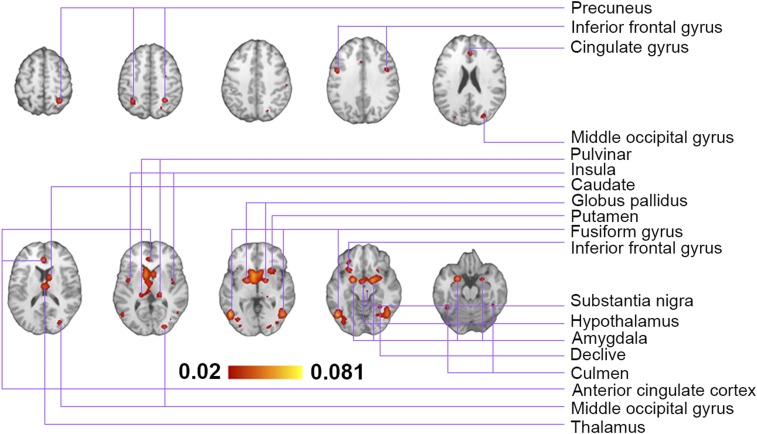
ALE analysis suggested that exposure to erotic media, in contrast to neutral stimuli such as sport activities or landscapes, induce significant activations in a number of cortical and subcortical structures.

**Fig. 4.**
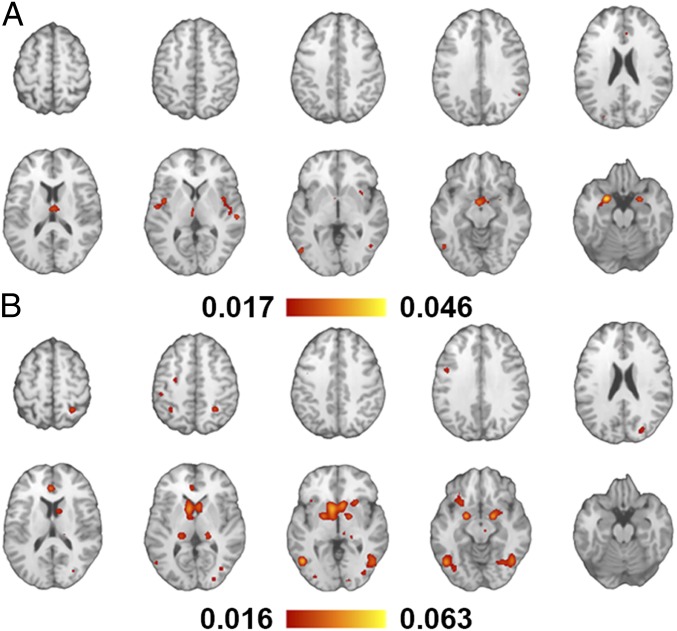
Neural correlates of sexual arousal cluster into 2 distinct patterns associated only to the stimulus type, namely video (*A*) and pictures (*B*).

Exposure to visual sexual stimuli, in contrast to presentation of sexually neutral figures such as sport activity and landscapes, was consistently reported to induce robust significant activations of insula, middle and inferior occipital and fusiform gyrus, amygdala, caudate, claustrum, globus pallidus, pulvinar, and substantia nigra (Fig. 3 and *SI Appendix*, Table S2). This arousal network can be clustered into 2 distinct components based on stimulus type (Fig. 4 *A* and *B*). Experiments using video and pictures as medium reported comparable number of coordinates (51% and 49% of all coordinates, respectively); however, watching erotic pictures leads to a more widespread activation pattern in the brain. A plausible explanation is the level of attention to nonsexual elements of visual presentations since the frequency of frame presentation in videos is on average much higher than the exposure time to pictures. Combined eye tracking and fMRI in future studies may shine light on this matter. Moreover, the preprocessing of images and the statistical treatment of the fMRI time series applied for analyzing data acquired during picture presentations may be suboptimal for capturing all features related to video presentation, which in turn may lead to irrelevant differences. Since the metaanalysis does not have access to raw data, we cannot evaluate this hypothesis within the scope of this study.

**Fig. 5.**
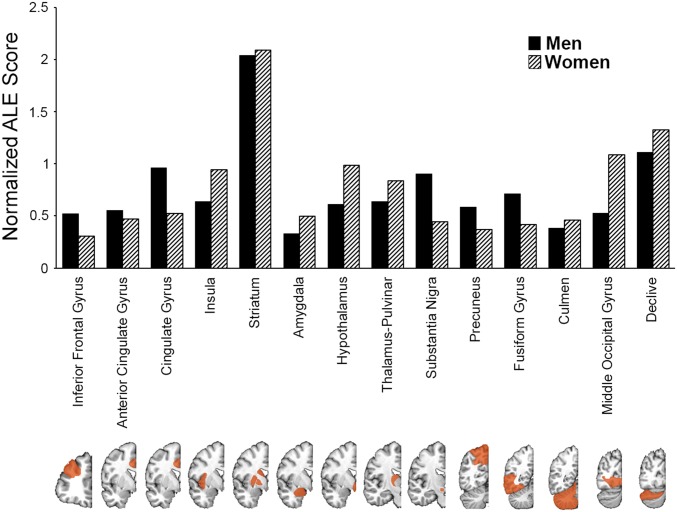
Erotic media stimulate numerous cortical and subcortical regions, including insula, middle occipital, anterior cingulate and fusiform gyrus, amygdala, striatum, pulvinar, and substantia nigra. In agreement with the cluster analysis (*Upper* plot), all regions (marked as orange masks on coronal slices of T1 anatomical image from anterior to posterior) respond to sexual stimuli in both men and women, albeit to different degrees as reflected by the normalized ALE scores. For consistent representation of ALE findings, the scores were normalized within each group since the ALE scores are calculated for each subgroup (e.g., men or women) separately from histograms associated with internal data distribution within the respective group. Larger ALE scores correspond to a stronger effect and as *P* values of a statistical test are uniquely obtained from distribution.

Two-step cluster analysis and ALE contrast analysis both suggest that there are no significant differences in neuronal correlates of sexual arousal between men and women (Fig. 5). Indeed, biological sex is the least relevant predictor (with less than 1% predictive power) for the clustering and classification of the data under investigation. These findings are robust with respect to the choice of *P* values for statistical analysis (*SI Appendix*, Tables S3–S6), which in turn suggests that the number of studies included in the metaanalysis for each biological sex is sufficient for reliable statistical conclusions.

As sexual orientation of study participants is a weak classifier (15.1% predictive power), it is critical to investigate whether biological sex may matter within such subgroups. The bisexual and transsexual groups contained small sample sizes such that the reliability of the metaanalysis with respect to these groups is limited (*SI Appendix*, Tables S7 and S8) and were not further analyzed. However, it is noteworthy that the ALE metaanalyses suggest that presentation of visual sexual stimuli induces activations in left cingulate gyrus (Brodmann area [BA] 31/32) in all groups. In addition, significant responses could be obtained bilaterally in middle occipital gyrus (BA 18/19) and superior temporal gyrus (BA 22/41), as well as unilaterally in left lingual gyrus (BA 18), right fusiform gyrus (BA 37), right precentral gyrus (BA 6), and right supramarginal gyrus (BA 40) among heterosexuals, homosexuals, and bisexuals. Homosexuals exposed to preferred sexual stimuli show significant bilateral responses in caudate body, cuneus, cerebellar-vermian declive, lingual and fusiform gyrus, and unilateral responses in right hemisphere, particularly in inferior and posterior parietal gyrus and inferior occipital gyrus (*SI Appendix*, Table S9). In contrast, heterosexuals show in agreement with previous study (17) lateralized left hemisphere activation (*SI Appendix*, Fig. S2 and Tables S10 and S11) and the involvement of subcortical areas such as thalamus and hypothalamus that have been hypothesized to show structural correlations (in terms of differences in size) with the sexual orientation of individuals (18). Contrary to the aforementioned differences between subjects of different sexual orientation, our present ALE metaanalysis of coordinates associated with stimulus-induced brain activation in female and male homo- and heterosexuals showed no significant sex-specific clusters in either group. It follows, that in contrast to common beliefs and reports of previous studies, our analysis demonstrates that there is no functional dimorphism in response to visual sexual stimuli between men and women. Varying permutation levels did not alter the outcome, supporting the robustness of the results.

### Links between Sexual Arousal and Brain Structure.

To date, only a single study (19) has investigated differences in brain structure associated with sexual arousal and desire, comparing gray matter volume (GMV) and white matter (WM) fractional anisotropy (FA) in females with hypoactive sexual desire disorder and a control healthy group. One research article, per se, does not permit a metaanalysis. However, the findings of this study indicate that sexual arousal and interest may correlate with GM volume particularly in insula of the right hemisphere and the anterior cingulate gyrus, both reported to show functional response to sexual stimuli. Therefore, the systematic review of sex differences in gray matter volume was focused on insula and anterior cingulate (ACC). In a Gaussian-process regression coordinate-based metaanalysis including 16 voxel-based morphometry (VBM) studies published until 2013, Ruigrok et al. (10) reported larger gray matter volumes in women in over 20 brain regions within particularly amygdala, anterior cingulate gyrus, and right insular cortex. However, more recent metaanalyses (11) at least partly contradict these findings by reporting a lack of sexual dimorphism in amygdala. We therefore conducted an independent and up-to-date (i.e., until April 2019) systematic review of original research articles to investigate whether there are any sex differences in gray matter volume of those brain structures. Out of 228 studies suggested by keyword-based search, 37 publications were potentially relevant. Thirty-six were original research articles in the English language that reported voxel-based sex differences in human brain. Among these studies, 7 provided statistical comparisons of gray matter volume between men and women in insula, anterior cingulate cortex, or both, of 3,723 adult (48.6 ± 9.8 y) healthy heterosexual study participants (20–26). No study included sexual arousal in its design. Furthermore, the studies did not provide the measured volume values (in milliliters) for men and women, instead coordinates for the significant regions as well as F/t scores or Z values were provided (*SI Appendix*, Table S12). It is noteworthy that Pletzer et al. (23) reported a larger ACC (x = −7, y = 63, z = 9 mm in MNI [Montreal Neurological Institute] coordinates) volume in women using contraception than in men but did not observe significant differences in the same region if women did not use contraceptives. The remaining 29 studies did not report any significance with respect to sex in GMV in these regions, while the data from the regions were analyzed and differences in volumes in other regions were determined. While all studies that report sex differences in insula and ACC suggest larger volumes in women than men, the majority (approximately 80%) of investigations did not reveal any differences in these regions. It can only be speculated that the lack of sexual dimorphism at the functional level is paralleled with a lack of dimorphism at the structural level. However, the systematic review does not permit any reliable conclusions on a causal relationship between structural features and functional response to visual sexual stimuli.

## Discussion

Erotic pictures and videos are widely assumed to induce differential response due to sexual duality (7). In particular, men are presumed to respond more strongly to visual sexual stimuli than do women (6). Previous studies indicate differences in how human brain in healthy (27, 28) and disease-states (29, 30) may process information in women and men. However, potential sex-specific neurobiological mechanisms underlying the observation of sexual content are not fully elucidated (31, 32). Sex differences were usually inferred with respect to sexual arousal (33) and sexual motivation (7, 34, 35). Earlier research was ambiguous with some researchers identifying differences regarding activated brain areas (36, 37), while others could only show small sex differences (38), if any at all. The assumed sex difference in the neural processing of sexual stimuli might have been due to various factors, including hormonal status, opposing attitudes toward sexual material, differentially pronounced arousal, varying levels of sexual motivation, or simply due to insufficient sample sizes. For instance, Hamann et al. (36) identified amygdala as a critical region that responds differently to sexual stimuli in 28 men and women. However, our metaanalysis with region-of-interest (ROI) response coordinates obtained from 1,850 individuals does not support their observation. While amygdala is a key region responsive to visual sexual stimuli, there are no significant differences at whole-brain level between the sexes.

Previous metaanalyses (39–41) either focused only on neuronal networks in heterosexual men or relied on limited sample sizes while considering a mixture of sensory modalities, including visual, tactile, and olfactory sexual stimulation and used data from different scanning modalities such as fMRI and PET for the analysis (42). Indeed the identification of sex-specific activation of subcortical brain regions such as hypothalamus in the recent study by Poeppl et al. (42) is most likely due to the inclusion of studies using penile and clitoral stimulation and exposure to male and female pheromones. In contrast, our metaanalysis has a wider scope but stricter inclusion criteria. Thereby, it challenges the common theories that sexual arousal differs between genders (7), which are largely based on subjective rating of sexual arousal and desire in response to sexual stimuli (6) instead of relying on measurable biological dimensions.

Evidence from studies examining habituation to sexual stimuli also offers evidence that men and women evaluate sexual stimuli using different strategies. Repeated exposure to sexually explicit slides of men and women typically produces both physiological and subjective habituation of sexual arousal in men (43), but inconsistent results in women.

Several limitations warrant attention and suggest directions for future research. First, the present metaanalysis was necessarily limited to functional neuroimaging experiments. In particular, lack of reporting of null results, raw data, and volume levels (in milliliters) for regional gray matter does not allow us to conduct a reliable metaanalysis of structural features that potentially relate to functional patterns of sexual arousal. Furthermore, the spatiotemporal scale of consideration does not allow any interpretations of the underlying synaptic and molecular mechanisms. Second, because the application of neuroimaging methods to investigate cue reactivity is a rapidly evolving field, standards for data acquisition and processing varied among the studies. For instance, the heterogeneity in the setup and paradigms of task-fMRI experiments as well as the low number of studies do not allow a complementary analysis with respect to attentional interference and other relevant factors. The quality scores of the reviewed studies (44) also suggest that the quality of reporting the methodological details of the included experiments varied considerably. While our sensitivity analysis does not show any significant impact of presentation paradigm (block design vs. event related), other methodological factors may still have an influence on the results. Unfortunately, it was not possible to test the moderating effects of all variables because of the low number of studies within methodological categories and/or lack of reporting the details, but these effects merit future investigation.

In conclusion, the present study provides comprehensive metaanalytic evidence that the neurocircuitries associated with sexual arousal do not differ in men and women independent of their sexual orientation. Visual sexual stimuli induce activation in the same cortical and subcortical regions in both men and women, while the limited sex differences that have been found and reported previously refer to subjective rating of the content. Therefore, our study contributes to the evidence-based characterization of sexual behavior and improves our understanding of neurobiological processes underlying response to sexual stimuli and arousal.

## Materials and Methods

The metaanalysis and systematic review were conducted as previously described (17) and reported according to the PRISMA (45) guidelines, i.e., evidence-based, standardized checklists determining the minimum set of items required in a review conducting metaanalysis, and assessing the overall quality of the body of evidence in previous papers and reviews. Details on search strategy, study selection, quality assessment, and data extraction are presented in *SI Appendix*.

### Outcomes and Effect Modifiers.

For the ALE metaanalysis, the primary outcome was the stereotaxic coordinates of brain regions responding significantly to erotic visual stimuli. If the coordinates of activated brain areas were not provided, then the congruent brain regions were identified within the Talairach Daemon Atlas (46) and the coordinates were calculated a posteriori as the center of mass (centroid) coordinates of the corresponding atlas regions. As mentioned above, the primary outcome of structural metaanalysis was mean ± SD volume of the brain regions of interest. Since these data were inconsistently reported, if at all, the primary outcome of the systematic review was the stereotaxic coordinates of voxel clusters that showed significant differences in GMV between men and women. Age, sex, sexual orientation, the so-called Kinsey rating scale for assessment of sexual orientation—typically ranging from 0, meaning exclusively heterosexual, to 6, meaning exclusively homosexual—handedness, stimulus presentation paradigm (block design or event related), analysis type (whole brain analysis vs. region-of-interest approach), and machine power (i.e., strength of magnetic field) were considered as effect modifiers.

### Metaanalyses of Functional Neuroimaging Observations.

The metaanalyses were conducted with the revised version (12, 13) of the ALE approach (47), using the GingerALE (v2.3.6) software package (available at http://brainmap.org/ale). ALE assesses the overlap between coordinates of significant neural responses reported in different studies by modeling them as spatial probability (Gaussian) distributions centered at the respective coordinates. To enable localization of results in BAs, which are currently defined only in the Talairach space, coordinates from studies reported in MNI space were transformed to Talairach space using the icbm2tal algorithm (48). To restrict metaanalytic results to gray matter, ALE analyses were constrained by a gray matter mask applied to extracted data, and thresholded at a voxel-wise *P* < 0.05 corrected for false discovery rate, with clusters ≥200 mm^3^. The ALE analyses were compared with relaxed clusters thresholded at an uncorrected voxel-wise *P* < 0.01 and ≥200 mm^3^. Peaks of the resulting clusters were labeled by reference to the Talairach Daemon Atlas, and all clusters were displayed on a single-subject Talairach template. Contrast analysis was conducted to combinatorically compare ALE datasets in a pairwise manner, in particular with respect to sexual orientation and sex. Permutations (10, 100, 1,000, and 2,000) were performed to correct for study sizes (12) and obtain a voxel-wise *P* value image showing where the true data’s values sit on the distribution of values in that voxel. Figures were generated using Mango (v4.0.1) software. The results of the metaanalyses are reported in tables containing information about the identified clusters. The clusters are ordered by their volume in descending order. For each cluster, the comprising brain regions, the designated BA (if given), weighted center in x, y, and z dimensions in Talairach space, the submaxima of the ALE scores and cluster volumes are provided. In addition, the ALE scores were normalized such that the significance and contribution of each cluster within a test can be presented by a probability measure (“relative importance”). Relative importance is calculated as the cumulative ALE scores with a cluster normalized to the sum of overall ALE scores for clusters with a volume larger than 200 mm^3^.

### Statistical Analysis of ALE Findings.

To assess the impact of inclusion of any partially nonindependent study on the results, jackknife analyses were conducted iteratively. Each partially nonindependent study was excluded and the density statistics of the significant clusters were recalculated. Subsequently, χ^2^ test or Fisher’s exact test was performed between the original and the leave-one-out recalculated density statistics. Since no individual study affected the overall statistics, the presented results are based on all studies. Moreover, regression analyses were performed to investigate the relationship between the number of significant clusters (as a topological measure of neuronal response patterns) and moderating variables given by study specifics such as the sample size and the number of reported foci. In addition, OFAT (one-factor-at-a-time) sensitivity analyses were performed a posteriori to ensure the robustness of the metaanalysis results with respect to biological (sex, sexual orientation, age, and handedness) as well as experimental (stimulus type and paradigm) factors. Therefore, ALE was applied on the proportion of studies within each demographic and experimental subgroup and differences between the proportions were tested between groups. In addition to the ALE contrast analysis, a 2-step hierarchical clustering algorithm using IBM SPSS statistics software (v24.0.0) was applied, which identifies groupings of categorical and continuous variables by running preclustering first and then by running hierarchical methods. Sexual orientation, presentation paradigm, stimulus type, brain regions, and hemispheres were treated as categorical variables, whereas the percentage of male participants in a study, their mean age and machine power were continuous numerical variables. The distance was given by the Log-likelihood measure, which places a probability distribution on the variables. Continuous variables are assumed to be normally distributed, while categorical variables are assumed to be multinomial. All variables are assumed to be independent. The Bayesian information criterion (BIC) was used to choose the number of clusters according to the intrinsic complexity of the data. The refined results of the clustering are presented as a hierarchical tree (*SI Appendix*, Fig. S2).

## Supplementary Material

Supplementary File

Supplementary File
