# Supplementary material for: Neural substrates of sexual arousal are not sex dependent
Source: Proc Natl Acad Sci U S A. 2019 Jul 15;116(31):15671–6. doi: 10.1073/pnas.1904975116 (PMC6681749; doi:10.1073/pnas.1904975116)
Supplement: Supplementary File [file pnas.1904975116.sapp.pdf]

Supplementary Information

Neural substrates of sexual arousal are not sex-dependent

Ekaterina Mitricheva, Rui Kimura, Nikos Logothetis, Hamid R. Noori

Max Planck Institute for Biological Cybernetics, Max Planck Ring 8, 72076 Tübingen, Germany

www.pnas.org/cgi/doi/10.1073/pnas.1904975116

Report of data mining process for meta-analysis and systematic review according to PRISMA guidelines

Search Strategy: The online portal of the National Library of Medicine (http://www.ncbi.nlm.nih.gov/pubmed/) including PubMed, PubMed Central and MEDLINE was used as the platform for literature research. A systematic screening of the original research articles published until January 2019 was performed based on the keywords: *heterosexual (OR) homosexual (OR) bisexual (OR) transsexual (OR) MRI (OR) visual (OR) fMRI (OR) sexual (OR) stimulus (OR) stimuli.* For the systematic review related to structural sex differences, the research article screening was based on the following keywords similar to previous meta-analyses (1): *(Insula OR anterior cingulate cortex/gyrus OR ACC) AND MRI AND grey AND matter AND (sex OR gender OR (male AND female) OR (males AND females)).* In addition, the reference sections of identified papers as well as review and meta-analysis articles were then screened for further relevant citations.

Study Selection: Two reviewers (EM and HRN) independently screened titles and abstracts of articles and reviewed the full text of any title or abstract deemed potentially eligible by either reviewer. Reviewers resolved disagreements by discussion. Among these studies, only peer-reviewed original research articles in English language were chosen for data mining. For the meta-analysis of functional response patterns to visual sexual stimuli studies were included if they provided analysis of brain responses (i.e. exact coordinates or explicit identification of the reactive brain regions) to visual stimuli (i.e. pictures and videos) either within a group with self-identified sexual orientation of the same or different sex, or between such individuals and subjects with a different sexual orientation. For the systematic review of sex differences in grey matter volume in insula and anterior cingulate, data was extracted only from studies that provided coordinates for the regions of interest as well as the results of statistical tests in terms of t- or Z-levels. If raw or corrected (with respect to total brain volume) mean ± SD volume were provided, then the study was also selected for meta-analysis. All participants were un-medicated healthy volunteers with no Axis I disorders. Axis I disorders, as defined in the Diagnostic and Statistical Manual (DSM-IV), include all psychological diagnostic categories, barring mental retardation and personality problems. Additional

exclusion criteria for all groups were: lifetime diagnosis of schizophrenia or psychotic episodes; diagnosis of manic disorder, obsessive compulsive disorder, alcohol use disorders, substance dependent disorder or post-traumatic stress disorder; treatment for mental disorders other than pathological gambling in the past 12 months; use of psychotropic medication; history or current treatment for neurological disorders, major internal disorders, brain trauma, or exposure to neurotoxic factors. Resting-state fMRI and other scanning modalities were excluded. Abstracts and unpublished studies were not included. Authors were contacted if critical information was missing or only partially provided in their articles.

Quality Assessment: Pairs of reviewers independently assessed the risk of bias and the reporting quality of individual studies by means of the 83-item checklist adapted from the guidelines for reporting an fMRI study (2). A minimum score of 15 out of 83 was assumed as an inclusion criterion.

Data Extraction: The following variables and parameters were extracted from relevant publications into a standardized template and used for further analysis:

1. **Publication Variables:** PubMed Identification Number (PMID), first author, year of publication, country;
2. **Demographic Variables:** Number of study participants, mean and standard deviation of the age, percentage of male individuals, sexual orientation, Kinsey scale, handedness, medications history, and in case of comparative studies number of participants in opposite group, and matching criteria;
3. **Experimental Variables:** Scanning modalities (i.e. machine power, number of channels per RF head coil, data type e.g. T2*-weighted/ T1-weighted/ T2-weighted, spatiotemporal resolution, and in case of structural data: voxel-based morphometry etc.), stimulus type (picture or video), contrast stimulus type (e.g. couples engaged in non-sexual activity, sport activity, landscapes), cue presentation paradigm (block-design or event-related);

Analysis Parameters: Analysis software and version, statistical analysis (whole brain or ROI-based), coordinates of activated brain regions (in Montreal Neurological Institute (MNI) or Talairach coordinates), if provided mean ± SD volume of the brain regions and the level of activation/deactivation.

Figure S1. Detailed flow diagram of study selection according to PRISMA guidelines.

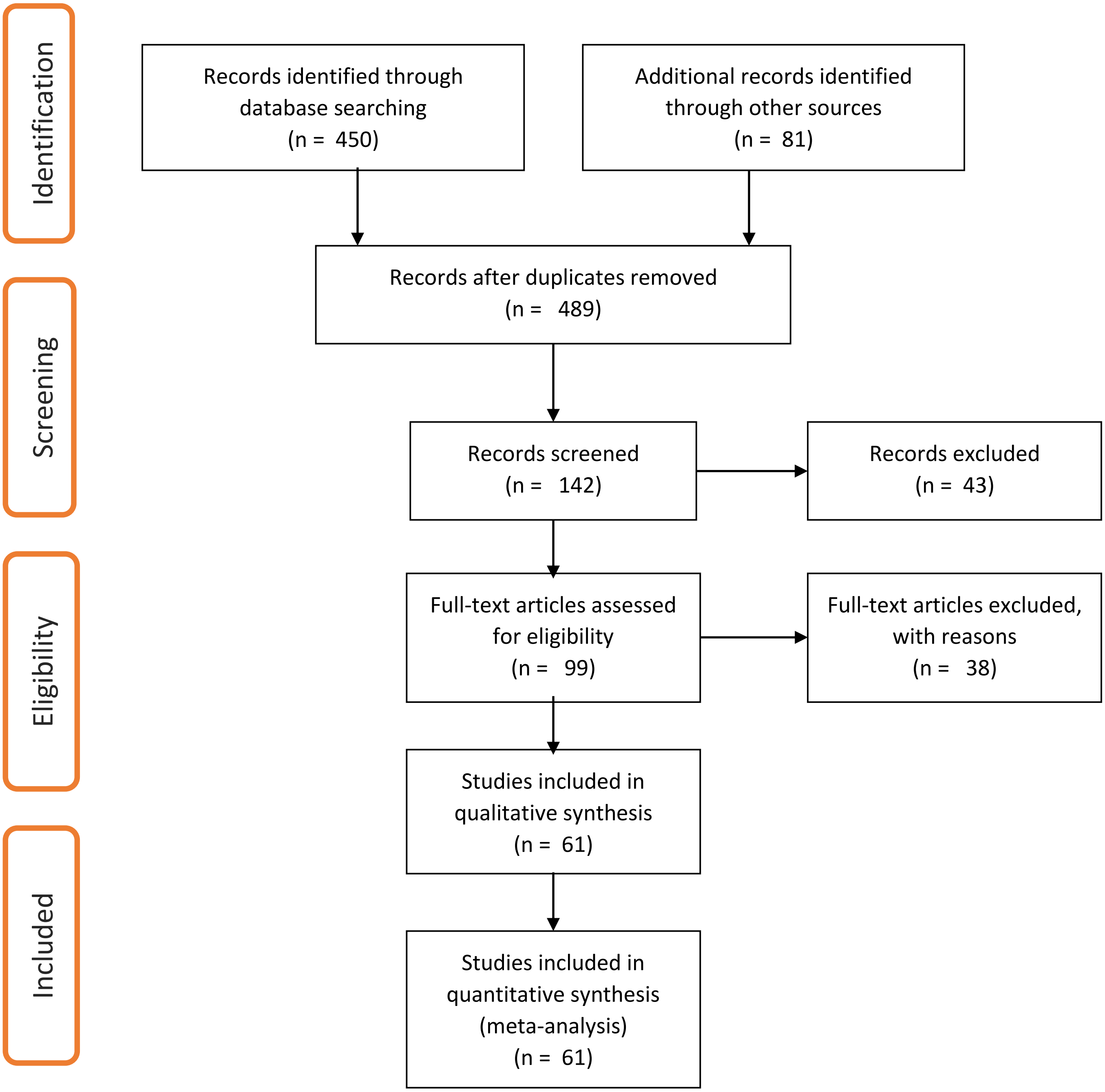

Figure S2. Axial presentation of the differences in neural response patterns between heterosexuals and homosexuals. Visual sexual stimuli induce a larger lateralized neuronal activation towards the left hemisphere in heterosexual in comparison with homosexual individuals. The significant clusters are colored according to their ALE score ranging from 0.013 to 0.042.

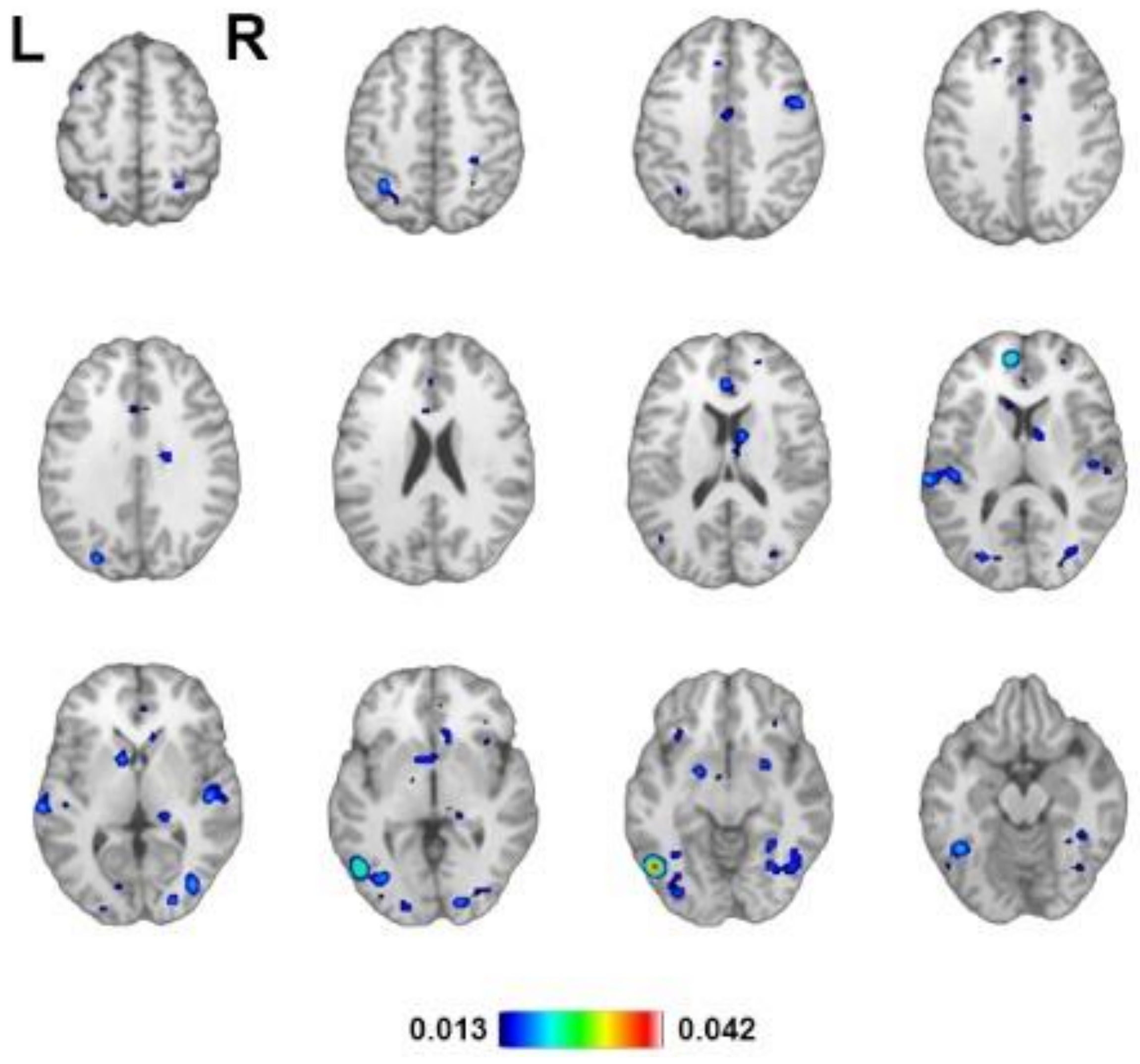

Figure S3. The tree-based classification model of the data. It classifies cases into groups or predicts values of a dependent (target) variable based on values of independent (predictor) variables. Two step clustering algorithm suggests stimulus/cue type (picture or video) as the predictor of maximal importance. Thus, the decision tree is based on cue type as main predictor.

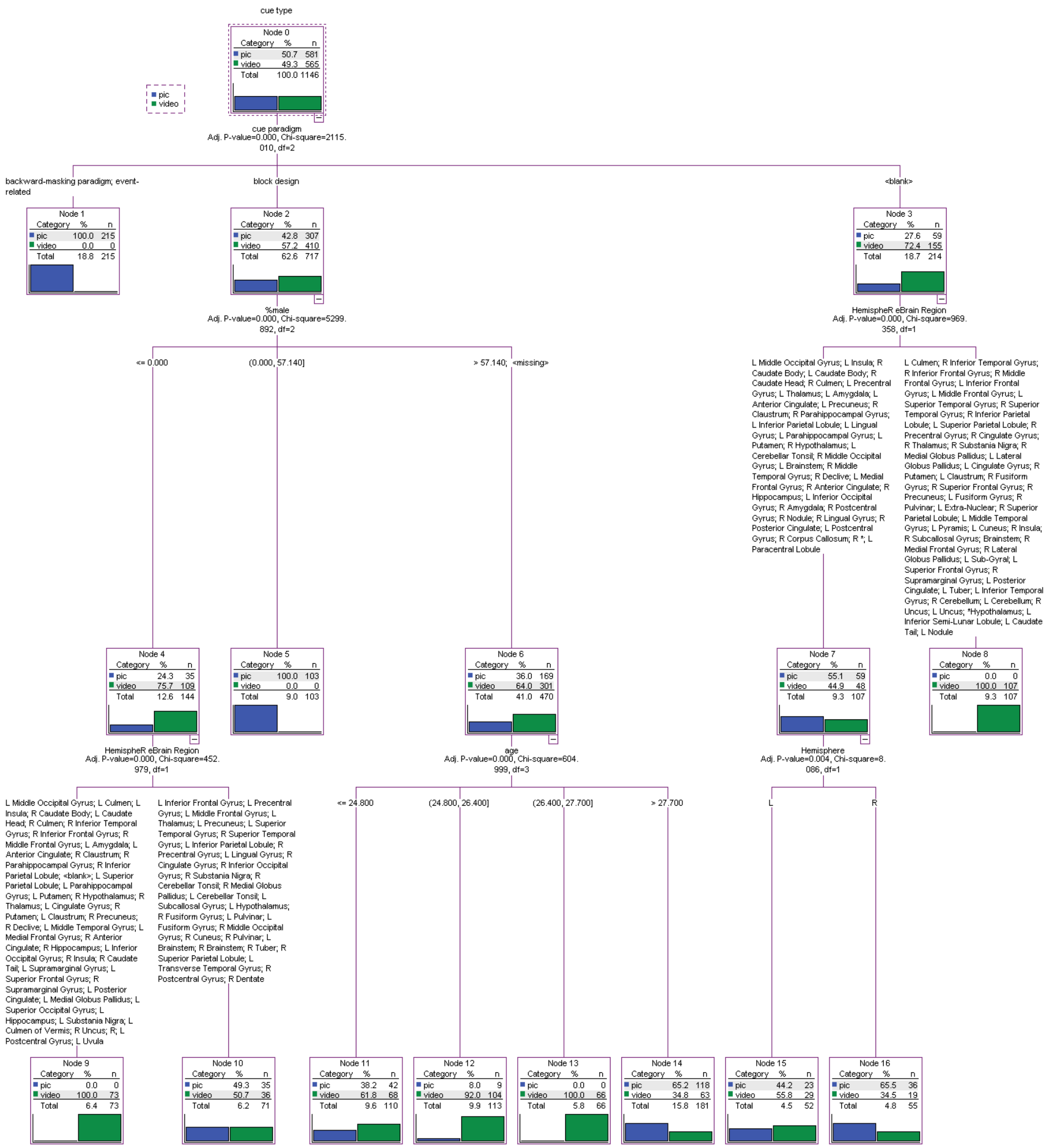

Table S1. Overall estimates of the included studies. In relation to transsexual individuals, FTM and MTF refer to female (F) to male (M) and male to female transformation, respectively. Duplicates are excluded from the number of study participants (n).

Groups	Sex	# Studies	n	Mean Age ± SD (y)
**Bisexual**	F	Safron et al. 2018 (3)	21	30.3 ± 6.4
	M	Safron et al. 2017 (4)	19	37.5 ± 6.4
**Heterosexual**	F	Safron et al. 2018 (3); Strahler et al. 2018 (5); Borg et al. 2014a,b (6, 7); Wehrum-Osinsky et al. 2014 (8); Kim et al. 2013 (9); Sylva et al. 2013 (10); Wehrum et al. 2013 (11); Woodard et al. 2013 (12); Demos et al. 2012 (13); Gillath et al. 2012 (14); Bianchi-Demicheli et al. 2011 (15); Arnow et al. 2009 (16); Klucken et al. 2009 (17); Savic and Lindstrom 2008 (18); Walter et al. 2008 (19); Sabatinelli et al. 2007 (20); Ponseti et al. 2006 (21); Stark et al. 2005 (22); Hamann et al. 2004 (23); Sabatinelli et al. 2004 (24); Karama 2002 (25)	560	26.0 ± 4.0
	M	Unterhorst et al. 2018 (26); Strahler et al. 2018 (5); Jordan et al. 2018 (27); Safron et al. 2017 (4); Wernicke et al. 2017 (28); Brand et al. 2016 (29); Seok et al. 2016 (30); Graf et al. 2014 (31); Oei et al. 2014 (32); Kühn and Gallinat, 2014 (33); Mascaro et al. 2014 (34); Wehrum-Osinsky et al. 2014 (8); Costumero et al. 2013 (35); Kim et al. 2013 (9); Wehrum et al. 2013 (11); Oei et al. 2012 (36); Kagerer et al. 2011 (37); Zhang et al. 2011 (38); Hu et al. 2011 (39); Seo et al. 2010 (40); Sundaram et al. 2010 (41); Metzger et al. 2010 (42); Gizewski et al. 2009 (43); Klucken et al. 2009 (17); Ponseti et al. 2009 (44); Seo et al. 2009 (45); Brunetti et al. 2008 (46); Bühler et al. 2008 (47); Hu et al. 2008 (48); Savic and Lindstrom 2008 (18); Paul et al. 2008 (49); Schiffer et al. 2008 (50); Walter et al. 2008 (19); Sabatinelli et al. 2007 (20); Safron et al. 2007 (51); Gizewski et al. 2006 (52); Kim et al. 2006 (53); Moulier et al. 2006 (54); Ponseti et al. 2006 (21); Ferretti et al. 2005 (55); Stark et al. 2005 (22); Hamann et al. 2004 (23); Sabatinelli et al. 2004 (24); Mouras et al. 2003 (56); Arnow et al. 2002 (57); Karama et al. 2002 (25); Beauregard et al. 2001 (58); Park et al. 2001 (59)	1054	28.5 ± 6.6
**Homosexual**	F	Safron et al. 2018 (3); Savic et al 2008 (18); Ponseti et al. 2006 (21)	55	28.0 ± 1.9
	M	Safron et al. 2017 (4); Hu et al. 2011 (39); Zhang et al. 2011 (38); Hu et al. 2008 (48); Savic and Lindstrom 2008 (18); Paul et al. 2008 (49); Ponseti et al. 2006 (21)	109	30.9 ± 3.2
**Transsexual**	FTM	Kim et al. 2016 (60)	11	41.5 ± 5.5
	MTF	Oh et al. 2012 (61); Gizewski et al. 2009 (43)	21	37.9 ± 2.6
**Overall**			1850	29.4 ± 6.0

Table S2. Brain regions associated with sexual arousal. Healthy individuals exposed to erotic pictures and videos show accentuated neural activation patterns in contrast to neutral visual stimulation. In particular, ALE meta-analysis (corrected for false discovery rate pID <0.05) suggests activations of insula, middle and inferior occipital and fusiform gyrus, amygdala, caudate, claustrum, globus pallidus, pulvinar, and substantia nigra. Coordinates are presented in Talairach space. For clusters with sub-peaks, primary peak is bolded and sub-peaks are italicized. Clusters with relative importance less <5% are shaded. While these clusters have a volume larger than 200 mm^3, their cumulative ALE scores are outside the 95% quantile.

Anatomical Region	Weighted Center (x,y,z)			Brodmann Area	Volume (mm^3)	ALE score	Relative Importance (%)
**L Lateral Globus Pallidus**	-1.3	-1.2	-2.3		23944	0.071458	29.1
L Caudate Head						0.071275	
R Medial Globus Pallidus						0.063293	
R Caudate Head						0.060864	
L Amygdala						0.055921	
L Thalamus						0.047616	
R Caudate Body						0.042929	
L Pulvinar						0.030309	
L Pulvinar						0.024629	
L Substantia Nigra						0.024496	
**L Middle Occipital Gyrus**	-42.8	-67.4	-6.6	37	7656	0.080538	10.1
L Inferior Occipital Gyrus				19		0.036159	
L Culmen						0.028757	
L Middle Occipital Gyrus				18		0.026369	
**R Fusiform Gyrus**	42.8	-62.3	-7.5	37	6144	0.056005	7.1
R Fusiform Gyrus				37		0.032246	
R Declive						0.031727	
**R Lingual Gyrus**	25.6	-83.6	9.8	17	2664	0.041032	5.7
R Middle Occipital Gyrus				19		0.032548	
R Lingual Gyrus				17		0.022856	
**R Precuneus**	27.1	-55.6	48.2	7	2440	0.053713	7.3
R Precuneus				7		0.023799	
R Precuneus				7		0.023032	
R Precuneus				7		0.022488	
**L Anterior Cingulate**	-0.9	33.5	12.6	24	2248	0.047023	2.8
**L Claustrum**	-27.4	19.7	-9.7		2104	0.035937	6.5
L Inferior Frontal Gyrus				47		0.028184	
L Inferior Frontal Gyrus				47		0.025215	
L Insula				13		0.021274	
**R Claustrum**	26	13.8	-5.3		1832	0.039797	2.3
**L Superior Parietal Lobule**	-30.3	-54.2	45.3	7	1440	0.036688	3.4
L Precuneus				7		0.020758	
**R Pulvinar**	16.6	-30.4	3.1		1256	0.034267	3.3
R Thalamus						0.021186	

**L Inferior Frontal Gyrus**	-45.7	1.1	30.4	6	1056	0.038055	2.2
**R Inferior Occipital Gyrus**	37.3	-78.8	-0.1	19	696	0.025747	1.5
**L Insula**	-39.6	-3	4.3	13	672	0.031169	1.8
**R Inferior Frontal Gyrus**	44	3.3	28.2	9	640	0.030416	1.8
**R Insula**	39.5	-4.4	3.9	13	472	0.023476	2.7
**R Insula**				13		0.02307	
**R Culmen**	30.3	-50.4	-11.6		432	0.030895	1.8
**L Cuneus**	-26.2	-81.6	22.8	19	408	0.027583	1.6
**L Cingulate Gyrus**	-2.9	18	27.1	24	392	0.02577	1.5
**L Inferior Parietal Lobule**	-44.7	-33.2	40.8	40	352	0.026356	1.6
**R Middle Frontal Gyrus**	30.2	-9.5	48.3	6	304	0.027036	1.6
**R Substantia Nigra**	9.8	-22	-9.1		296	0.024097	2.7
R Substantia Nigra						0.021254	
**R Postcentral Gyrus**	55.7	-24.8	35.5	2	200	0.025426	1.5

Table S3. Regions in which sexual cues induced significantly different activation responses than contrasted neutral cues in male participants. *$p < .05$, corrected, voxelwise, for the false discovery rate. ALE = activation likelihood estimate. Coordinates are in Talairach space. For clusters with sub-peaks, primary peak is bolded and sub-peaks are italicized.

Anatomical Region	Weighted Center (x,y,z)			Brodmann Area	Volume (mm^3)	ALE score	Relative Importance (%)
**L Amygdala**	-5.1	-0.7	-1		11224	0.047921382	38.5
L Thalamus						0.042276394	
L Caudate Head						0.039614864	
R Caudate Head						0.038639594	
L Amygdala						0.036768116	
L Hypothalamus						0.03509055	
R Caudate Body						0.027035099	
L Amygdala						0.020057363	
**R Inferior Temporal Gyrus**	43.8	-59.2	-8.4	19	2824	0.032368105	8.4
R Fusiform Gyrus				37		0.03047994	
**L Middle Occipital Gyrus**	-47.3	-65.5	-4.3	37	2816	0.048047878	11.1
R Amygdala	22.7	-1.3	-12.2		1840	0.035074487	
**R Lingual Gyrus**	24	-87.5	2.4	17	952	0.03782408	5.1
L Culmen	-41.7	-50.7	-18.7		880	0.027779741	3.7
**L Inferior Parietal Lobule**	-30.3	-52	43.4	40	808	0.027705273	3.7
**R Superior Parietal Lobule**	27.3	-52.4	46.9	7	712	0.030980863	4.1
**L Insula**	-40.2	-3.6	5.4	13	648	0.029987777	4.0
**L Inferior Occipital Gyrus**	-33.8	-73.6	-4.8	19	560	0.025050608	3.4
**R Insula**	31.6	13.4	-6.1	13	424	0.020162899	2.7
**L Inferior Parietal Lobule**	-45.5	-33	39.3	40	368	0.024424419	3.3
**L Cuneus**	-25.3	-90.6	2.2	18	280	0.022239197	3.0
**R Postcentral Gyrus**	55.8	-25.1	35.9	2	240	0.02440132	3.3
**R Anterior Cingulate**	1.6	32.2	17.4	24	232	0.021947337	2.9
**L Inferior Frontal Gyrus**	-47.3	4	29	9	200	0.021348292	2.9

Table S4. Regions in which sexual cues induced significantly different activation responses than contrasted neutral cues in female participants. **p* < .05, corrected, voxelwise, for the false discovery rate. ALE = activation likelihood estimate. Coordinates are in Talairach space. For clusters with sub-peaks, primary peak is bolded and sub-peaks are italicized.

**Anatomical Region**	**Weighted Center (x,y,z)**			**Brodmann Area**	**Volume (mm^3)**	**Extrema Value**	**Relative Importance (%)**
**L Fusiform Gyrus**	-46.5	-66.1	-9.6	19	408	0.025985	27.2
**R Declive**	30.6	-65.5	-11.4		384	0.024251	25.3
**L Amygdala**	-20.9	0.1	-18.6		360	0.023228	24.3
**L Precentral Gyrus**	-45	-0.9	29.9	6	248	0.022212	23.2

Table S5. Regions in which sexual cues induced significantly different activation responses than contrasted neutral cues in male participants. Relaxed $p < .01$, uncorrected, voxelwise. ALE = activation likelihood estimate. Coordinates are in Talairach space. For clusters with sub-peaks, primary peak is bolded and sub-peaks are italicized.

Anatomical Region	Weighted Center (x,y,z)			Brodmann Area	Volume (mm^3)	Extrema Value	Relative Importance (%)
**L Amygdala**	1.2	1.6	-1.3		30912	0.047921	34.4
L Thalamus						0.042276	
L Caudate Head						0.039615	
R Caudate Head						0.03864	
L Amygdala						0.036768	
L Hypothalamus						0.035091	
R Amygdala						0.035074	
R Caudate Body						0.027035	
R Anterior Cingulate				24		0.021947	
L Substania Nigra						0.02178	
R Insula				13		0.020163	
L Amygdala						0.020057	
R Caudate Head						0.018996	
L Anterior Cingulate				24		0.017096	
R Anterior Cingulate				24		0.016765	
R Anterior Cingulate				24		0.015814	
L Pulvinar						0.014819	
**L Middle Occipital Gyrus**	-43.4	-63.9	-7.6	37	9336	0.048048	7.4
L Culmen						0.02778	
L Inferior Occipital Gyrus				19		0.025051	
**R Inferior Temporal Gyrus**	42.7	-60.5	-7.3	19	7168	0.032368	7.5
R Fusiform Gyrus				37		0.03048	
R Middle Occipital Gyrus				19		0.021093	
R Culmen						0.017845	
**R Lingual Gyrus**	25	-85.8	5.5	17	2720	0.037824	5.1
R Middle Occipital Gyrus				19		0.017955	
R Cuneus				19		0.014014	
**L Inferior Parietal Lobule**	-28.4	-53.4	43.9	40	2688	0.027705	4.4
L Precuneus				7		0.019235	
L Superior Parietal Lobule				7		0.013809	
**L Insula**	-41.2	-5.1	3.9	13	1968	0.029988	3.4
L Superior Temporal Gyrus				22		0.016637	
**R Parahippocampal Gyrus**	19.2	-22.9	-8.5	35	1936	0.019631	4.2
R Parahippocampal Gyrus				35		0.019019	
R Thalamus						0.018146	
**R Superior Parietal Lobule**	27.6	-52.4	45.9	7	1760	0.030981	2.3
**R Middle Frontal Gyrus**	43.1	3.1	34.2	6	1688	0.017586	3.5

R Inferior Frontal Gyrus				9		0.015845	
R Inferior Frontal Gyrus				9		0.014833	
**L Insula**	-34.6	16.8	-7	13	1512	0.021049	4.0
L Inferior Frontal Gyrus				47		0.018876	
L Extra-Nuclear				13		0.015262	
**L Cuneus**	-26.5	-89.7	1.7	18	1392	0.022239	1.6
**L Inferior Parietal Lobule**	-46.8	-32.6	38.8	40	1312	0.024424	1.8
**L Inferior Frontal Gyrus**	-45	2.8	30.5	9	1256	0.021348	2.7
L Precentral Gyrus				6		0.015735	
**R Insula**	39	-2.4	2.5	13	984	0.02084	2.6
R Claustrum						0.014883	
**R Postcentral Gyrus**	55.6	-25.4	35.7	2	752	0.024401	1.8
**R Cingulate Gyrus**	1.3	-8.5	38.5	24	696	0.01916	1.4
**L Cuneus**	-25.6	-81.4	25	19	536	0.022288	1.6
**L Anterior Cingulate**	-14.1	36	3.7		472	0.016645	1.2
**R Nodule**	0.8	-47	-32.6		464	0.019786	1.4
**R Precuneus**	20.9	-69.2	42.7	7	464	0.017063	1.2
**L Inferior Frontal Gyrus**	-24.8	30.2	-11	47	328	0.015729	1.2
**R Pulvinar**	16.2	-27.3	15.9		296	0.019004	1.4
**L Medial Frontal Gyrus**	-7.4	38.1	-14.1	11	272	0.016948	1.2
**L Middle Temporal Gyrus**	-43.5	-67.6	17.3	39	272	0.017951	1.3
**R Caudate Body**	15	23.6	10.1		240	0.016361	1.2

Table S6. Regions in which sexual cues induced significantly different activation responses than contrasted neutral cues in female participants. Relaxed *p* < .01, uncorrected, voxelwise. ALE = activation likelihood estimate. Coordinates are in Talairach space. For clusters with sub-peaks, primary peak is bolded and sub-peaks are italicized.

Anatomical Region	Weighted Center (x,y,z)			Brodmann Area	Volume (mm^3)	Extrema Value	Relative Importance (%)
**L Amygdala**	-17.8	0.4	-12.2		6096	0.023228	11.5
L Caudate Head						0.014976	
L Putamen						0.014933	
L Medial Globus Pallidus						0.014092	
Hypothalamus						0.012619	
L Inferior Frontal Gyrus				47		0.010018	
L Putamen						0.009215	
**R Declive**	32.8	-61.7	-10.3		4896	0.024251	8.6
R Declive						0.018973	
R Fusiform Gyrus				37		0.017041	
R Inferior Temporal Gyrus				37		0.013412	
**L Anterior Cingulate**	-4.7	41.1	12.4	24	3136	0.018261	6.1
L Medial Frontal Gyrus				10		0.017215	
L Anterior Cingulate				24		0.01706	
**L Fusiform Gyrus**	-45.3	-64.4	-9.9	19	2408	0.025985	5.3
L Declive						0.010554	
L Declive						0.00904	
**R Middle Occipital Gyrus**	26.5	-79.2	17.9	19	1912	0.01817	4.1
R Middle Occipital Gyrus				19		0.016638	
**R Medial Globus Pallidus**	14.2	-4.6	-8.1		1432	0.016132	3.4
R Parahippocampal Gyrus				34		0.01277	
**L Precentral Gyrus**	-45.4	-0.9	30.6	6	1360	0.022212	2.6
**R Inferior Occipital Gyrus**	38	-79.9	-2.6	19	1056	0.01693	2.0
**R Superior Parietal Lobule**	30.5	-54.3	54.1	7	1040	0.018536	2.2
**L Inferior Occipital Gyrus**	-34.7	-82.1	-8.1	18	984	0.015102	3.2
L Middle Occipital Gyrus				18		0.01245	
**R Inferior Frontal Gyrus**	43.3	2.3	27.6	9	944	0.018094	2.1
**R Insula**	47	-14.3	5.8	13	928	0.013525	3.0
R Superior Temporal Gyrus				22		0.012021	
**R Inferior Frontal Gyrus**	33	6.9	-14	47	872	0.012579	2.8
**R Superior Temporal Gyrus**				38		0.011043	
**L Precuneus**	-30	-63	37.2	7	864	0.010695	4.6
L Superior Parietal Lobule				7		0.009996	
L Angular Gyrus				39		0.009965	
L Middle Temporal Gyrus				39		0.008836	
**L Parahippocampal Gyrus**	-39.3	-30.1	-6.4	36	728	0.015787	1.8
**R Middle Frontal Gyrus**	29.7	-9.3	46.9	6	704	0.014209	1.7

**R Pulvinar**	14.7	-27.2	5.9		688	0.015783	2.9
R Inferior Parietal Lobule				40		0.009163	
**L Cingulate Gyrus**	-2.1	17.2	27.4	32	624	0.011723	2.4
R Anterior Cingulate				24		0.008873	
**L Superior Temporal Gyrus**	-61.4	-27.8	6.8	42	560	0.015704	2.9
L Superior Temporal Gyrus				41		0.008975	
**L Substania Nigra**	-11.5	-23.2	-11.9		488	0.011965	2.5
L Parahippocampal Gyrus				35		0.009697	
**L Lingual Gyrus**	-9.5	-78.4	-1.4	18	480	0.016565	1.9
**L Inferior Parietal Lobule**	-58	-29	31	40	448	0.015293	1.8
**R Precuneus**	21.1	-72.3	34.3	19	432	0.015318	1.8
**R Thalamus**	10.4	-15.9	14.9		408	0.011492	2.4
R Thalamus						0.008713	
**R Supramarginal Gyrus**	51.5	-53.4	32.9	40	368	0.012415	1.4
**R Uncus**	19.9	0	-21.9	28	352	0.012511	1.5
**R Substania Nigra**	8	-22	-10.4		328	0.012413	1.4
**L Thalamus**	-3.4	-13.1	7.4		304	0.00949	3.2
L Thalamus						0.008968	
L Thalamus						0.008905	
**L Middle Frontal Gyrus**	-26.4	-10.4	46	6	304	0.011852	1.4
**L Superior Temporal Gyrus**	-49.7	-11.8	3.9	22	296	0.011732	1.4
**L Transverse Temporal Gyrus**	-41.5	-28.5	9.5	41	296	0.011241	1.3
**R Inferior Occipital Gyrus**	14	-92	-6.4	17	288	0.011489	1.3
**L Cingulate Gyrus**	-2.6	-22.6	34	24	256	0.010929	1.3
**L Middle Occipital Gyrus**	-26.7	-80.5	11.9	19	232	0.01033	1.2
**R Putamen**	24.9	3.8	1.2		200	0.010045	1.2

Table S7. Regions in which erotic visual stimulation (pictures and videos) induced significantly different responses than contrasted cues among bisexual study participants. Coordinates are in Talairach space. For clusters with sub-peaks, primary peak is bolded and sub-peaks are italicized.

Anatomical Region	Weighted Center (x,y,z)			Brodmann Area	Volume (mm^3)	Extrema Value
**R Sub-Gyral Parietal Lobe**	30.1	-41.1	44.6		3544	0.012177
R Inferior Parietal Lobule						0.009981
R Precuneus				7		0.008566
R Precuneus				7		0.008564
**R Supramarginal Gyrus**	48.1	-58.5	32.8	40	2264	0.009437
R Middle Temporal Gyrus				39		0.00918
R Inferior Parietal Lobule				39		0.008735
R Superior Temporal Gyrus	53	-19	4.7	41	1864	0.008335
R Superior Temporal Gyrus				22		0.008193
R Superior Temporal Gyrus				22		0.008082
R Superior Temporal Gyrus				22		0.007959
**R Middle Occipital Gyrus**	27.4	-79.2	15.9	19	1680	0.01375
R Middle Occipital Gyrus				19		0.012356
**L Superior Temporal Gyrus**	-48.3	-13.7	4.1	22	1184	0.008911
L Superior Temporal Gyrus				22		0.00876
**R Declive**	29.3	-52.1	-11.8		464	0.008444
**L Precentral Gyrus**	-24	-19.1	57.9	4	464	0.008312
**R Declive**	32.3	-66.3	-10.7		448	0.00841
**R Precentral Gyrus**	26	-16.6	56.5	6	448	0.008442
**L Middle Occipital Gyrus**	-37.1	-80.2	-10.4	18	440	0.008321
**R Inferior Occipital Gyrus**	37.6	-78.3	-3.3	19	440	0.008357
**R Middle Occipital Gyrus**	29.1	-87.4	3.8	18	440	0.008229
**L Superior Temporal Gyrus**	-53.8	-25.2	8.3	41	440	0.008216
**R Cingulate Gyrus**	12.4	-52.9	25.6	31	432	0.008245
**L Inferior Parietal Lobule**	-35	-62.2	37.3	39	432	0.008128
**L Superior Parietal Lobule**	-32.5	-52.6	51.9	7	432	0.008424

**L Inferior Occipital Gyrus**	-23.3	-89	-8	18	424	0.008201
**L Lingual Gyrus**	-9.5	-78.3	-1.4	18	424	0.008365
**L Declive**	-23.3	-79.7	-15.3		416	0.008238
**L Middle Occipital Gyrus**	-29	-84	0.4	18	416	0.008231
**R Middle Occipital Gyrus**	23.9	-81.1	-1.1	18	416	0.008175
**L Cingulate Gyrus**	-1.4	-25.3	33.3	31	416	0.008202
**R Fusiform Gyrus**	43.3	-60.8	-9.8	37	408	0.008225
**L Superior Temporal Gyrus**	-42.8	-22.8	8.7	13	408	0.007894
**L Angular Gyrus**	-48.9	-64.8	34.1	39	408	0.008153
**L Inferior Parietal Lobule**	-35.1	-40.9	50.2	40	408	0.008024
**R Lingual Gyrus**	12.8	-94.7	-8.1	17	400	0.008267

Table S8. Regions in which erotic visual stimulation (pictures and videos) induced significantly different responses than contrasted cues among transsexual study participants. Coordinates are in Talairach space. For clusters with sub-peaks, primary peak is bolded and sub-peaks are italicized.

Anatomical Region	Weighted Center (x,y,z)			Brodmann Area	Volume (mm^3)	Extrema Value
**L Putamen**	-26.4	-8.6	-4.4		3336	0.006951
L Amygdala						0.006914
L Lateral Globus Pallidus						0.006377
**R Hypothalamus**	-1.5	-0.3	-0.1		2968	0.006995
L Caudate Head						0.006279
L Caudate Head						0.006161
**R Caudate Body**	13.6	2.9	15.9		1544	0.006277
R Caudate Body						0.006269
**R Claustrum**	29.1	8.1	1.1		1032	0.006561
**R Cingulate Gyrus**	2.7	20.5	28.7	32	1032	0.006648
**R Amygdala**	22.5	-2	-11		1008	0.006698
**R Hippocampus**	23.5	-39.5	-1.5		1000	0.006792
**L Thalamus Medial Dorsal Nucleus**	-5.6	-13.2	10.6		992	0.00615
**L Cingulate Gyrus**	-11.2	13.6	36.6	32	992	0.006097
**R Cerebellar Tonsil**	9	-47.1	-34.5		984	0.005979
**L Caudate Body**	-18.8	-15.7	22.8		984	0.006088
**L Substania Nigra**	-13.5	-17.6	-11.8		976	0.006268
**R Parahippocampal Gyrus**	9.7	-38	9	30	960	0.006154
**R Claustrum**	34	-10.9	5.9		928	0.006216

Table S9. Regions in which erotic visual stimulation (pictures and videos) induced significantly different responses than contrasted cues among homosexual study participants. Coordinates are in Talairach space. For clusters with sub-peaks, primary peak is bolded and sub-peaks are italicized. Clusters with relative importance less <5% are shaded.

Anatomical Region	Weighted Center (x,y,z)			Brodmann Area	Volume (mm^3)	ALE Extrema Value	Relative Importance
**L Middle Occipital Gyrus**	-42.1	-67	-8.8	37	3080	0.018343	12.82
L Inferior Occipital Gyrus				19		0.009292	
L Fusiform Gyrus				37		0.008545	
L Inferior Occipital Gyrus				18		0.008522	
L Declive						0.008402	
L Fusiform Gyrus				37		0.006892	
**R Superior Temporal Gyrus**	49.2	-19.7	6.7	13	1696	0.013117	8.41
R Insula				13		0.009015	
R Superior Temporal Gyrus				41		0.00878	
R Superior Temporal Gyrus						0.008443	
L Superior Temporal Gyrus	-49.9	-28	6.1	41	1560	0.013436	6.71
L Middle Temporal Gyrus				22		0.009074	
L Superior Temporal Gyrus				42		0.008893	
**R Postcentral Gyrus**	29	-38.8	46.6	3	1360	0.015611	6.66
R Inferior Parietal Lobule				40		0.008847	
R Postcentral Gyrus				5		0.006681	
R Fusiform Gyrus	44	-60.9	-7.2	37	952	0.009207	5.26
R Fusiform Gyrus				19		0.008555	
R Middle Occipital Gyrus				37		0.006857	
**R Nodule**	-2.2	-46.6	-31		944	0.011283	4.23
L Cerebellar Tonsil						0.008523	
**R Culmen**	29.7	-49.3	-11.6		864	0.013309	2.84
**L Cuneus**	-25.4	-81.6	24.9	19	760	0.01277	2.73
**L Middle Occipital Gyrus**	-32.7	-77.5	7.4	19	664	0.009403	3.77
L Middle Occipital Gyrus				18		0.008228	
**L Superior Temporal Gyrus**	-56.5	-15	2.3	22	592	0.008597	3.58
L Superior Temporal Gyrus				22		0.008148	
**R Precentral Gyrus**	27.5	-10.2	52.9	6	424	0.0085	3.61
R Sub-Gyral Frontal Lobe				6		0.008389	
**R Inferior Occipital Gyrus**	37.6	-81.2	-1	19	336	0.008821	1.89
**L Middle Occipital Gyrus**	-23.5	-93.3	7.4	18	264	0.008428	1.80

**L Cingulate Gyrus**	-23.8	-37.3	39.9	31	256	0.008838	1.89
**L Precuneus**	-21.3	-60.4	48.5	7	256	0.008411	1.80
**R Anterior Cingulate**	4.7	40.4	-3.6	32	248	0.008564	1.83
**L Superior Parietal Lobule**	-29.6	-52	49.3	7	248	0.008652	1.85
**R Inferior Parietal Lobule**	56.8	-45.3	35.1	40	232	0.008187	1.75
**R Culmen**	-1	-54	-18		224	0.008433	1.80
**L Lingual Gyrus**	-12.5	-78.8	3.9	18	224	0.008374	1.79
**R Cuneus**	29.4	-76.6	10	30	224	0.008188	1.75
**R Anterior Cingulate**	18.3	27.3	14.2	32	224	0.008362	1.79
**L Caudate Body**	-20.6	-13.4	26		224	0.008287	1.77
**L Lingual Gyrus**	-18	-92.2	-2.7	17	216	0.008277	1.77
**R Lingual Gyrus**	29.5	-67.9	5.7	19	216	0.008268	1.77
**L Caudate Body**	-9.5	19.7	7.9		216	0.008406	1.80
**R Caudate Body**	15.2	-13.4	26.8		216	0.008253	1.76
**R Lingual Gyrus**	15.5	-89.3	-4.8	17	208	0.008018	1.71
**R Supramarginal Gyrus**	54	-53	26.2	40	208	0.008314	1.78
**R Superior Parietal Lobule**	20.2	-64	54.3	7	208	0.008529	1.82
**R Precentral Gyrus**	31.6	-11.1	59.8	6	208	0.008268	1.77
**R Declive**	29.7	-66	-13.3		200	0.008244	1.76
**R Inferior Parietal Lobule**	37.3	-31.6	38.9	40	200	0.008058	1.72

Table S10. Regions in which erotic visual stimulation (pictures and videos) induced significantly different responses than contrasted cues among heterosexual study participants. Coordinates are in Talairach space. For clusters with sub-peaks, primary peak is bolded and sub-peaks are italicized. Clusters with relative importance less <5% are shaded.

Anatomical Region	Weighted Center (x,y,z)			Brodmann Area	Volume (mm^3)	ALE Extrema Value	Relative Importance
**L Lateral Globus Pallidus**	-12	-1.8	-5.7		6984	0.025848	10.32%
L Anterior Cingulate				25		0.020094	
L Lateral Globus Pallidus						0.019862	
L Hypothalamus						0.014821	
**L Anterior Cingulate**	-2.2	19.2	25.6	24	4136	0.027938	7.02%
L Cingulate Gyrus				32		0.01523	
L Medial Frontal Gyrus				9		0.011701	
**R Medial Globus Pallidus**	24.6	0.7	-5.8		4032	0.018982	12.65%
R Putamen						0.018413	
R Claustrum						0.014189	
R Insula				13		0.013846	
R Parahippocampal Gyrus				34		0.012696	
R Putamen						0.011387	
R Uncus				34		0.009357	
**R Thalamus Anterior Nucleus**	6.7	-6.6	14.3		2136	0.017197	3.99%
R Thalamus Medial Dorsal Nucleus						0.014021	
**L Middle Occipital Gyrus**	-47.9	-65.3	-6.2	37	2064	0.025041	4.52%
L Inferior Temporal Gyrus				37		0.010269	
**R Middle Occipital Gyrus**	28.9	-81.9	2.7	19	1840	0.016963	5.53%
R Middle Occipital Gyrus				18		0.016054	
R Middle Occipital Gyrus				18		0.010216	
**L Medial Frontal Gyrus**	-5.9	50	8.6	10	1160	0.020771	2.66%
**L Inferior Frontal Gyrus**	-32.7	19.2	-12.4	47	1064	0.016455	3.94%
L Inferior Frontal Gyrus				47		0.014365	
**R Caudate Head**	9.9	18.7	-0.4		1040	0.016194	2.07%
**L Culmen**	-40.2	-52.2	-18.7		984	0.019781	2.53%
**R Middle Frontal Gyrus**	43.4	-0.2	39.7	6	920	0.015579	1.99%
**R Thalamus**	17.3	-31.8	0.2		792	0.014727	1.88%
**L Superior Temporal Gyrus**	-58.4	-27.4	7.6	42	776	0.013504	4.70%
L Superior Temporal Gyrus				22		0.012007	
L Superior Temporal Gyrus				41		0.011193	
**L Inferior Parietal Lobule**	-31.4	-56.5	42.8	7	720	0.016438	2.10%
**R Precuneus**	27.4	-53.8	49.4	7	656	0.014613	1.87%
**L Lingual Gyrus**	-30.9	-72	-5.2	18	576	0.015714	2.01%
**R Medial Frontal Gyrus**	23.4	47.2	12.3	10	528	0.0126	3.21%

R Superior Frontal Gyrus				10		0.012496	
**R Inferior Frontal Gyrus**	32	27.1	-5.6	47	496	0.012795	1.64%
**R Superior Temporal Gyrus**	60.5	-52.6	13.1	22	464	0.014865	3.04%
R Supramarginal Gyrus				40		0.008883	
**R Fusiform Gyrus**	40.7	-66	-12.4	19	336	0.01074	1.37%
**R Superior Temporal Gyrus**	48.7	-18.1	2.8	22	320	0.011945	1.53%
**L Middle Temporal Gyrus**	-42.8	-67.1	17	39	320	0.012808	1.64%
**R Precentral Gyrus**	23	-13.3	65.5	6	320	0.013695	1.75%
**L Middle Frontal Gyrus**	-36.7	8.6	53.2	6	304	0.013529	1.73%
**R Middle Temporal Gyrus**	47.7	-60.7	5.2	37	264	0.011431	1.46%
**R Caudate Body**	21.6	-14.5	28.6		248	0.011605	1.49%
**R Fusiform Gyrus**	39.3	-44.9	-14.5	37	232	0.012154	1.56%
**L Middle Frontal Gyrus**	-20.7	9.2	59.6	6	232	0.011485	1.47%
**L Anterior Cingulate**	-0.8	33.2	18.3	32	216	0.010404	1.33%
**R Cingulate Gyrus**	2	-4	42.8	24	208	0.010483	1.34%
**L Middle Occipital Gyrus**	-31.8	-82.1	-10.7	18	200	0.010795	1.38%
**L Thalamus Lateral Posterior Nucleus**	-17.1	-17.7	15.2		200	0.011536	1.48%
**R Medial Frontal Gyrus**	2.5	37.6	29	9	200	0.011035	1.41%
**R Postcentral Gyrus**	55.6	-21.6	33.5	2	200	0.01069	1.37%

Table S11. Regions in which erotic visual stimulation (pictures and videos) induced significantly stronger activation in heterosexuals than in homosexuals. Coordinates are in Talairach space. For clusters with sub-peaks, primary peak is bolded and sub-peaks are italicized. Clusters with relative importance less <5% are shaded.

Anatomical Region	Weighted Center (x,y,z)			Brodmann Area	Volume (mm^3)	ALE Score	Relative Importance (%)
**L Middle Occipital Gyrus**	-42	-65.1	-9.1	37	6528	0.042287	11.49
L Fusiform Gyrus				37		0.021754	
L Lingual Gyrus				18		0.01764	
L Middle Occipital Gyrus				18		0.015223	
L Declive						0.014738	
L Inferior Temporal Gyrus				37		0.010483	
**L Lateral Globus Pallidus**	-12	-1.7	-5.8		5520	0.025848	7.59
L Anterior Cingulate				25		0.020095	
L Lateral Globus Pallidus						0.019863	
L Hypothalamus						0.014821	
**L Anterior Cingulate**	-1.1	21.6	23	24	4840	0.027985	9.08
L Anterior Cingulate				24		0.016668	
L Cingulate Gyrus				32		0.015287	
L Cingulate Gyrus				32		0.013315	
L Medial Frontal Gyrus				9		0.011701	
R Anterior Cingulate				24		0.011596	
**R Middle Occipital Gyrus**	29.3	-81.1	3.3	19	3152	0.018849	6.16
R Lingual Gyrus				17		0.017129	
R Middle Occipital Gyrus				18		0.01616	
R Middle Occipital Gyrus				19		0.013286	
**R Medial Globus Pallidus**	24.1	0.8	-5.5		2720	0.018984	8.42
R Putamen						0.018413	
R Claustrum						0.014189	
R Insula				13		0.013846	
R Parahippocampal Gyrus				34		0.012696	
R Putamen						0.011387	
**R Thalamus**	7.8	-6.4	13.5		2400	0.019309	3.46
R Thalamus Medial Dorsal Nucleus						0.017437	
**L Superior Temporal Gyrus**	-54.4	-27.2	6.6	42	2376	0.020599	3.62
L Superior Temporal Gyrus				41		0.017829	
**R Declive**	38.7	-64.4	-9.2		1960	0.015524	4.15
R Fusiform Gyrus				19		0.01492	
R Fusiform Gyrus				37		0.013699	
**R Superior Temporal Gyrus**	49.7	-19	4.9	13	1760	0.01926	4.28
R Superior Temporal Gyrus						0.013315	
R Superior Temporal Gyrus				41		0.01293	

**L Inferior Parietal Lobule**	-28.5	-57.3	45.2	40	1352	0.017847	4.09
L Precuneus				7		0.014824	
L Superior Parietal Lobule				7		0.01079	
**L Medial Frontal Gyrus**	-6.6	49.6	8.5	10	1296	0.026735	2.52
**R Nodule**	-1.5	-45.9	-31.7		992	0.018206	2.89
L Cerebellar Tonsil						0.012562	
**R Precentral Gyrus**	44.2	-0.5	39	6	880	0.016193	1.52
**L Inferior Frontal Gyrus**	-32.8	19.5	-12.6	47	848	0.016497	2.91
L Inferior Frontal Gyrus				47		0.014462	
**R Culmen**	32.4	-48.7	-12.2		848	0.015621	2.71
R Fusiform Gyrus				37		0.013204	
**R Caudate Head**	9.8	18.6	-0.5		752	0.016195	1.52
**L Middle Occipital Gyrus**	-19.8	-79.8	7.2	19	744	0.015672	3.72
L Lingual Gyrus				18		0.01239	
L Cuneus				17		0.011452	
**R Cingulate Gyrus**	1.2	-8.1	38	24	704	0.01336	1.26
**R Thalamus**	17.4	-32.4	0.8		608	0.01495	1.41
**L Cuneus**	-26.3	-80.7	25.2	19	584	0.018596	1.75
**L Lingual Gyrus**	-18.8	-92.1	-1.1		544	0.013651	1.28
**R Caudate Body**	19.3	-14.4	27.6		480	0.014418	1.36
**R Precuneus**	27.1	-53.9	49.5	7	464	0.01464	1.38
**R Medial Frontal Gyrus**	23.2	47.5	12.6	10	320	0.0126	2.36
R Superior Frontal Gyrus				10		0.012496	
**R Superior Temporal Gyrus**	61	-53.2	12.8	22	304	0.014867	1.40
**R Inferior Frontal Gyrus**	31.9	27.4	-6	47	280	0.012795	1.20
**L Caudate Body**	-8.7	17.6	7.8		264	0.012747	1.20
**R Postcentral Gyrus**	27.5	-37.7	43.5	3	264	0.01566	1.47
**R Precentral Gyrus**	23.2	-13.4	65.2	6	256	0.013846	1.30
**L Middle Frontal Gyrus**	-36.9	8.8	53	6	240	0.013529	1.27
**L Middle Temporal Gyrus**	-42.5	-67.4	16.9	39	216	0.012974	1.22

Table S12. Systematic review of studies reporting sex differences in grey matter volume (GMV) of insula (Ins) and anterior cingulate (ACC). Hereby, women (w) show larger GMV in Ins and ACC than men (m). These studies represent approximately 20% of structural investigations of regional GMV differences between healthy male and females. The majority of publications report inconsistent findings. Most publications do not report significant sex differences in the Ins and ACC, while differences in other brain areas are observed (see as an example (62)). Δ represents differences. w* means that this study identified differences between women on contraceptives and men but no differences between w and m could be observed for Ins and ACC. Coordinate, if reported in MNI, were transformed and are presented in Talairach-space.

**Publication**	**N(f)**	**Age (yrs)**	**Δ**	**Brain region**	**Cluster size (voxels)**	**x**	**y**	**z**	**t/F score**	**Z**
Lotze et al 2019 (63)	2838	52.4±13.7	w>m	R ACC	3087	8	41	6	9.22	
			w>m	L ACC	3087	-8	38	20	8.05	
			w>m	R pIns	349	36	-17	3	8.31	
			w>m	L pIns	199	-40	-15	.34	6.48	
Yang et al 2017 (64)	82(53)	27.7±8.0	w>m	R Ins	1380	38	12	-1	26.84	
			w>m	L Ins	772	-33	11	-1	20.63	
Lentini et al 2013 (65)	86(45)	35±7	w>m	ACC	1.6 cm^3	-16	19	-10		4.8
Paus et al 1996 (66)	105(42)	25.2±7.7		R ACC	F: 6.22 ccm<br>M: 5.81 ccm				4.86	
Pletzer et al 2010 (67)	28(14)	24.3±4.1	w*>m	L/R ACC	189502	-7	56	18	11.45	
Yamasue et al 2008 (68)	155(66)	28.5±4.3	w>m	L Ins	9160	-53	-22	16		4.04
				R Ins	11616	47	-22	21		3.89
				L ACC	64	-14	46	12		3.33
Chen et al 2007 (69)	411(227)	46.7±1.4	w>m	ACC, dorsal		-1	4	46	5.81	

REFERENCES

1. Marwha D, Halari M, & Eliot L (2017) Meta-analysis reveals a lack of sexual dimorphism in human amygdala volume. *Neuroimage* 147:282-294.
2. Poldrack RA, *et al.* (2008) Guidelines for reporting an fMRI study. *Neuroimage* 40(2):409-414.
3. Safron A, *et al.* (2018) Neural Correlates of Sexual Orientation in Heterosexual, Bisexual, and Homosexual Women. *Sci Rep* 8(1):673.
4. Safron A, *et al.* (2017) Neural Correlates of Sexual Orientation in Heterosexual, Bisexual, and Homosexual Men. *Sci Rep* 7:41314.
5. Strahler J, Kruse O, Wehrum-Osinsky S, Klucken T, & Stark R (2018) Neural correlates of gender differences in distractibility by sexual stimuli. *Neuroimage* 176:499-509.
6. Borg C, de Jong PJ, & Georgiadis JR (2014) Subcortical BOLD responses during visual sexual stimulation vary as a function of implicit porn associations in women. *Soc Cogn Affect Neurosci* 9(2):158-166.
7. Borg C, *et al.* (2014) Brain processing of visual stimuli representing sexual penetration versus core and animal-reminder disgust in women with lifelong vaginismus. *PLoS One* 9(1):e84882.
8. Wehrum-Osinsky S, *et al.* (2014) At the second glance: stability of neural responses toward visual sexual stimuli. *J Sex Med* 11(11):2720-2737.
9. Kim TH, Kang HK, & Jeong GW (2013) Assessment of brain metabolites change during visual sexual stimulation in healthy women using functional MR spectroscopy. *J Sex Med* 10(4):1001-1011.
10. Sylva D, *et al.* (2013) Neural correlates of sexual arousal in heterosexual and homosexual women and men. *Horm Behav* 64(4):673-684.
11. Wehrum S, *et al.* (2013) Gender commonalities and differences in the neural processing of visual sexual stimuli. *J Sex Med* 10(5):1328-1342.
12. Woodard TL, Nowak NT, Balon R, Tancer M, & Diamond MP (2013) Brain activation patterns in women with acquired hypoactive sexual desire disorder and women with normal sexual function: a cross-sectional pilot study. *Fertil Steril* 100(4):1068-1076.
13. Demos KE, Heatherton TF, & Kelley WM (2012) Individual differences in nucleus accumbens activity to food and sexual images predict weight gain and sexual behavior. *J Neurosci* 32(16):5549-5552.
14. Gillath O & Canterberry M (2012) Neural correlates of exposure to subliminal and supraliminal sexual cues. *Soc Cogn Affect Neurosci* 7(8):924-936.
15. Bianchi-Demicheli F, *et al.* (2011) Neural bases of hypoactive sexual desire disorder in women: an event-related FMRI study. *J Sex Med* 8(9):2546-2559.
16. Arnow BA, *et al.* (2009) Women with hypoactive sexual desire disorder compared to normal females: a functional magnetic resonance imaging study. *Neuroscience* 158(2):484-502.
17. Klucken T, *et al.* (2009) Neural activations of the acquisition of conditioned sexual arousal: effects of contingency awareness and sex. *J Sex Med* 6(11):3071-3085.
18. Savic I & Lindstrom P (2008) PET and MRI show differences in cerebral asymmetry and functional connectivity between homo- and heterosexual subjects. *Proc Natl Acad Sci U S A* 105(27):9403-9408.
19. Walter M, *et al.* (2008) Distinguishing specific sexual and general emotional effects in fMRI-subcortical and cortical arousal during erotic picture viewing. *Neuroimage* 40(4):1482-1494.
20. Sabatinelli D, Bradley MM, Lang PJ, Costa VD, & Versace F (2007) Pleasure rather than salience activates human nucleus accumbens and medial prefrontal cortex. *J Neurophysiol* 98(3):1374-1379.
21. Ponseti J, *et al.* (2006) A functional endophenotype for sexual orientation in humans. *Neuroimage* 33(3):825-833.
22. Stark R, *et al.* (2005) Erotic and disgust-inducing pictures--differences in the hemodynamic responses of the brain. *Biol Psychol* 70(1):19-29.

23. Hamann S, Herman RA, Nolan CL, & Wallen K (2004) Men and women differ in amygdala response to visual sexual stimuli. *Nat Neurosci* 7(4):411-416.
24. Sabatinelli D, Flaisch T, Bradley MM, Fitzsimmons JR, & Lang PJ (2004) Affective picture perception: gender differences in visual cortex? *Neuroreport* 15(7):1109-1112.
25. Karama S*, et al.* (2002) Areas of brain activation in males and females during viewing of erotic film excerpts. *Hum Brain Mapp* 16(1):1-13.
26. Unterhorst K*, et al.* (2018) An Exploratory Study on the Central Nervous Correlates of Sexual Excitation and Sexual Inhibition. *J Sex Res*:1-12.
27. Jordan K*, et al.* (2018) Sex attracts - neural correlates of sexual preference under cognitive demand. *Brain Imaging Behav* 12(1):109-126.
28. Wernicke M*, et al.* (2017) Neural correlates of subliminally presented visual sexual stimuli. *Conscious Cogn* 49:35-52.
29. Brand M, Snagowski J, Laier C, & Maderwald S (2016) Ventral striatum activity when watching preferred pornographic pictures is correlated with symptoms of Internet pornography addiction. *Neuroimage* 129:224-232.
30. Seok JW, Park MS, & Sohn JH (2016) Neural pathways in processing of sexual arousal: a dynamic causal modeling study. *Int J Impot Res* 28(5):184-188.
31. Graf H*, et al.* (2014) Erotic stimulus processing under amisulpride and reboxetine: a placebo-controlled fMRI study in healthy subjects. *Int J Neuropsychopharmacol* 18(2).
32. Oei NY, Both S, van Heemst D, & van der Grond J (2014) Acute stress-induced cortisol elevations mediate reward system activity during subconscious processing of sexual stimuli. *Psychoneuroendocrinology* 39:111-120.
33. Kuhn S & Gallinat J (2014) Brain structure and functional connectivity associated with pornography consumption: the brain on porn. *JAMA Psychiatry* 71(7):827-834.
34. Mascaro JS, Hackett PD, & Rilling JK (2014) Differential neural responses to child and sexual stimuli in human fathers and non-fathers and their hormonal correlates. *Psychoneuroendocrinology* 46:153-163.
35. Costumero V*, et al.* (2013) Reward sensitivity is associated with brain activity during erotic stimulus processing. *PLoS One* 8(6):e66940.
36. Oei NY, Rombouts SA, Soeter RP, van Gerven JM, & Both S (2012) Dopamine modulates reward system activity during subconscious processing of sexual stimuli. *Neuropsychopharmacology* 37(7):1729-1737.
37. Kagerer S*, et al.* (2011) Neural activation toward erotic stimuli in homosexual and heterosexual males. *J Sex Med* 8(11):3132-3143.
38. Zhang M*, et al.* (2011) Neural circuits of disgust induced by sexual stimuli in homosexual and heterosexual men: an fMRI study. *Eur J Radiol* 80(2):418-425.
39. Hu SH*, et al.* (2011) Haemodynamic brain response to visual sexual stimuli is different between homosexual and heterosexual men. *J Int Med Res* 39(1):199-211.
40. Seo Y, Jeong B, Kim JW, & Choi J (2010) The relationship between age and brain response to visual erotic stimuli in healthy heterosexual males. *Int J Impot Res* 22(4):234-239.
41. Sundaram T*, et al.* (2010) Time-course analysis of the neuroanatomical correlates of sexual arousal evoked by erotic video stimuli in healthy males. *Korean J Radiol* 11(3):278-285.
42. Metzger CD*, et al.* (2010) High field FMRI reveals thalamocortical integration of segregated cognitive and emotional processing in mediodorsal and intralaminar thalamic nuclei. *Front Neuroanat* 4:138.
43. Gizewski ER*, et al.* (2009) Specific cerebral activation due to visual erotic stimuli in male-to-female transsexuals compared with male and female controls: an fMRI study. *J Sex Med* 6(2):440-448.
44. Ponseti J*, et al.* (2009) Assessment of sexual orientation using the hemodynamic brain response to visual sexual stimuli. *J Sex Med* 6(6):1628-1634.

45. Seo Y, Jeong B, Kim JW, & Choi J (2009) Plasma concentration of prolactin, testosterone might be associated with brain response to visual erotic stimuli in healthy heterosexual males. *Psychiatry Investig* 6(3):194-203.
46. Brunetti M, *et al.* (2008) Hypothalamus, sexual arousal and psychosexual identity in human males: a functional magnetic resonance imaging study. *Eur J Neurosci* 27(11):2922-2927.
47. Buhler M, Vollstadt-Klein S, Klemen J, & Smolka MN (2008) Does erotic stimulus presentation design affect brain activation patterns? Event-related vs. blocked fMRI designs. *Behav Brain Funct* 4:30.
48. Hu SH, *et al.* (2008) Patterns of brain activation during visually evoked sexual arousal differ between homosexual and heterosexual men. *AJNR Am J Neuroradiol* 29(10):1890-1896.
49. Paul T, *et al.* (2008) Brain response to visual sexual stimuli in heterosexual and homosexual males. *Hum Brain Mapp* 29(6):726-735.
50. Schiffer B, *et al.* (2008) Functional brain correlates of heterosexual paedophilia. *Neuroimage* 41(1):80-91.
51. Safron A, *et al.* (2007) Neural correlates of sexual arousal in homosexual and heterosexual men. *Behav Neurosci* 121(2):237-248.
52. Gizewski ER, *et al.* (2006) There are differences in cerebral activation between females in distinct menstrual phases during viewing of erotic stimuli: A fMRI study. *Exp Brain Res* 174(1):101-108.
53. Kim SW, *et al.* (2006) Brain activation by visual erotic stimuli in healthy middle aged males. *Int J Impot Res* 18(5):452-457.
54. Moulier V, *et al.* (2006) Neuroanatomical correlates of penile erection evoked by photographic stimuli in human males. *Neuroimage* 33(2):689-699.
55. Ferretti A, *et al.* (2005) Dynamics of male sexual arousal: distinct components of brain activation revealed by fMRI. *Neuroimage* 26(4):1086-1096.
56. Mouras H, *et al.* (2003) Brain processing of visual sexual stimuli in healthy men: a functional magnetic resonance imaging study. *Neuroimage* 20(2):855-869.
57. Arnow BA, *et al.* (2002) Brain activation and sexual arousal in healthy, heterosexual males. *Brain* 125(Pt 5):1014-1023.
58. Beauregard M, Levesque J, & Bourgouin P (2001) Neural correlates of conscious self-regulation of emotion. *J Neurosci* 21(18):RC165.
59. Park K, *et al.* (2001) A new potential of blood oxygenation level dependent (BOLD) functional MRI for evaluating cerebral centers of penile erection. *Int J Impot Res* 13(2):73-81.
60. Kim GW, Kim SK, & Jeong GW (2016) Neural activation-based sexual orientation and its correlation with free testosterone level in postoperative female-to-male transsexuals: preliminary study with 3.0-T fMRI. *Surg Radiol Anat* 38(2):245-252.
61. Oh SK, *et al.* (2012) Brain activation in response to visually evoked sexual arousal in male-to-female transsexuals: 3.0 tesla functional magnetic resonance imaging. *Korean J Radiol* 13(3):257-264.
62. Savic I & Arver S (2011) Sex dimorphism of the brain in male-to-female transsexuals. *Cereb Cortex* 21(11):2525-2533.
63. Lotze M, *et al.* (2019) Novel findings from 2,838 Adult Brains on Sex Differences in Gray Matter Brain Volume. *Sci Rep* 9(1):1671.
64. Yang X, *et al.* (2017) Sex differences in the clinical characteristics and brain gray matter volume alterations in unmedicated patients with major depressive disorder. *Sci Rep* 7(1):2515.
65. Lentini E, Kasahara M, Arver S, & Savic I (2013) Sex differences in the human brain and the impact of sex chromosomes and sex hormones. *Cereb Cortex* 23(10):2322-2336.
66. Paus T, *et al.* (1996) In vivo morphometry of the intrasulcal gray matter in the human cingulate, paracingulate, and superior-rostral sulci: hemispheric asymmetries, gender differences and probability maps. *J Comp Neurol* 376(4):664-673.

67. Pletzer B, *et al.* (2010) Menstrual cycle and hormonal contraceptive use modulate human brain structure. *Brain Res* 1348:55-62.
68. Yamasue H, *et al.* (2008) Sex-linked neuroanatomical basis of human altruistic cooperativeness. *Cereb Cortex* 18(10):2331-2340.
69. Chen X, Sachdev PS, Wen W, & Anstey KJ (2007) Sex differences in regional gray matter in healthy individuals aged 44-48 years: a voxel-based morphometric study. *Neuroimage* 36(3):691-699.
